# An Evaluation of the Belief in Science Scale

**DOI:** 10.3389/fpsyg.2019.00861

**Published:** 2019-04-16

**Authors:** Neil Dagnall, Andrew Denovan, Kenneth Graham Drinkwater, Andrew Parker

**Affiliations:** Department of Psychology, Manchester Metropolitan University, Manchester, United Kingdom

**Keywords:** belief in science, psychometric validation, reality testing, thinking style, convergent validity

## Abstract

The Belief in Science Scale (BISS) is a unidimensional measure that assesses the degree to which science is valued as a source of superior knowledge. Due to increased academic interest in the concept of belief in science, the BISS has emerged as an important measurement instrument. Noting an absence of validation evidence, the present paper, via two studies, evaluated the scale’s factorial structure. Both studies drew on data collected from previous research. Study 1 (*N* = 686), using parallel analysis and exploratory factor analysis, identified a unidimensional solution accounting for 56.43% of the observed variance. Study 2 (*N* = 535), using an independent sample, tested the unidimensional solution using confirmatory factor analysis (CFA). Data-model fit was good (marginal for RMSEA): CFI = 0.93, TLI = 0.91, RMSEA = 0.09 (90% CI of 0.08 to 0.10), SRMR = 0.04. Invariance testing across gender supported invariance of form, factor structure, and item intercepts for this one-factor model. BISS at the overall level correlated negatively with the reality testing dimension of the Inventory of Personality Organization (IPO-RT), demonstrating convergent validity. Researchers often use the IPO-RT as an indirect index of preference for experiential processing (intuitive thinking). In this context, only BISS scores above the median (second quartile) produced a reduction in experiential-based thinking. The authors discuss these findings in the context of belief in science as a psychometric construct.

## Introduction

Beliefs are a fundamental aspect of human cognition that fulfill important individual and social functions. Explicitly, beliefs provide meaning, comfort, and communality (Hogg and Mulling, 1999; Heine et al., 2006). This is particularly true of religious faith, which is associated with a range of positive psychological benefits. These include moderating negative factors related to lack of control (Kay et al., 2009), reducing anxiety (Inzlicht et al., 2011) and decreasing stress (Ano and Vasconcelles, 2005). Farias et al. (2013) contend that secular beliefs, such as Humanism and political ideologies perform comparable functions within non-religious individuals (Gray, 2004).

Although science and religion offer competing, often contradictory explanations, at a deeper, conceptual level, research suggests that they perform comparable psychological functions (i.e., structure life, provide reassurance, and facilitate social integration) (Ziman, 1978/1991). In support of this notion, studies report that beliefs related to human advancement offer positive, compensatory psychological functions (Rutjens et al., 2009, 2010). Explicitly, higher levels of belief in science are associated with positive psychological outcomes, such as happiness, lower levels of stress and reduced death anxiety (Aghababaei et al., 2016).

Acknowledging the potentially important role that secular beliefs play in modern society, Farias et al. (2013) developed the Belief in Science Scale (BISS). The BISS is a 10-item research tool, which measures the degree to which individuals endorse the legitimacy of the scientific approach. Particularly, the BISS assesses belief in the value of science as an institution and a source of superior knowledge. Accordingly, the scale recognizes differences in attitudes toward science. These range from rejection of the scientific approach, through acceptance of science as a reliable but fallible source of knowledge, to the conviction that science provides exclusive, veridical insights into reality. The latter doctrinaire perspective depicts science as a unique, central value. Consistent with this, the defining features of belief in science are confidence and trust in the validity of scientific methods and outcomes. Furthermore, higher belief in science is associated with outright dismissal of notions that sit outside of the traditional scientific framework. This manifests typically as rejection of scientifically unsubstantiated beliefs (i.e., paranormal) and religious skepticism.

Farias et al. (2013) tested the notion that belief in science provides secular individuals with psychological meaning and comfort in threatening contexts by conducting two related studies. These necessitated the development of BISS. Prior to the first experiment, Farias et al. (2013) gave items assessing belief in science to a sample of 144 participants. Subsequent psychometric examination, in the form of exploratory factor analysis (varimax rotation), yielded a single dimension accounting for 57% of the variance. All items loaded (≥0.56) and the scale demonstrated high internal consistency (α = 0.86). The overall sample mean (*M* = 3.23, *SD* = 1.04) was consistent with moderate belief in science. In study two (*N* = 60), further consideration of the psychometric properties of BISS, also found good internal consistency (α = 0.88).

Following the initial evaluation, Farias et al. (2013) used the BISS in their experiments. The first, found that rowers in a high-stress condition (pre-completion) vs. low-stress condition (training) reported greater belief in science. This result was congruent with the notion that belief in science helps secular individuals cope with stress. Although, Farias et al. (2013) acknowledged that context manipulation (competition vs. training) might affect also scientific focus (i.e., encourage emphasis on training regimen and equipment).

Within the second experiment, participants were assigned randomly to one of two mortality salience conditions (thoughts and feeling about own death vs. experiencing dental pain; control) and completed self-report measures assessing scientific determinism (Paulhus and Carey, 2010), religiosity and affect (negative and positive) (Watson et al., 1988).

Noting potential construct overlap, a moderate positive correlation between belief in science and scientific determinism (Paulhus and Carey, 2010), Farias et al. (2013) conducted a principal components analysis (PCA) on all science-related items. This used oblimin rotation, an oblique solution that permits factor correlation. The PCA identified three related but distinct factors: belief in science, original 10-items (eigenvalue = 5.74, loadings ≥0.62); scientific determinism (environmental factors), 3-items (eigenvalue = 2.02, loadings ≥0.68); and scientific determinism (biological factors), 4-items (eigenvalue = 1.79, loadings ≥0.66). This outcome supported the supposition that belief in science, although correlated with scientific determinism, was a separate construct. Consistent with study one outcomes, analysis revealed that participants in the mortality salience condition (vs. controls) scored higher on belief in science.

Overall, findings were consistent with Farias et al.’s (2013) conceptualisation of science as a form of “faith” in secular individuals that facilitates coping in stressful and anxiety-provoking situations. Furthermore, Farias et al. (2013) concluded that analytical thinking, rational enquiry and consideration of empirical evidence were key characteristics associated with scientific thinking. In this context, belief in science places an emphasis on fact based, objective (vs. objective experiential) evidence.

The BISS has also demonstrated criterion validity across a range of studies. For instance, Irwin et al. (2016) reported a negative moderate correlation (*r* = -0.55) between belief in science and the New Age Beliefs subscale of the Survey of Scientifically Unsubstantiated Beliefs (SUBS) (Irwin and Marks, 2013). This was consistent with Irwin et al. (2015), who observed strong negative associations between BISS and SUBS subscales (New Age Beliefs, *r* = -0.63; Traditional Religious Beliefs, *r* = -0.71). Moreover, Irwin et al. (2015) reported a moderate negative relationship (*r* = -0.32) between BISS and The Inventory of Personality Organization (IPO–RT; Lenzenweger et al., 2001). The IPO–RT assesses self-reported proneness to deficits in reality testing and researchers often use the scale as an index of experiential, intuitive thinking style (Drinkwater et al., 2012; Dagnall et al., 2015a, 2018; Denovan et al., 2017b).

Consistent with this notion, Irwin et al. (2016) found that believers in the paranormal tended to discount the values of science, and preferred to endorse ideas based on their emotional (rather than their rational) appeal. Accordingly, believers subject decisions to less critical scrutiny. Irwin et al. (2016) concluded that these characteristics reflect opposing worldviews. The scientific perspective comprises presumptive skepticism and an acceptance of the values of science, whereas a subjective and anti-materialistic outlook on life typifies paranormal belief (Zusne and Jones, 1989). Generally, these findings concur with preceding work that indicates that faith in science, religion and the paranormal represent independent dimensions of belief (Williams et al., 1989; Ståhl et al., 2016).

Despite these encouraging outcomes, the BISS is psychometrically underdeveloped. Even though widely cited, researchers have yet to validate the BISS. Indeed, consideration of the literature reveals that other than the reported EFA, the BISS structure remains unsubstantiated. Furthermore, within studies employing the BISS, authors have either failed to include psychometric details (Valdesolo et al., 2016), or merely confirmed that the BISS possesses high internal consistency (i.e., Irwin et al., 2015, α = 0.93; Ståhl et al., 2016, α = 0.96). This lacks exactitude and rigor because scale analysis has failed to progress beyond EFA. Hence, further research is required to evaluate the measurement properties of the BISS.

Additionally, EFA is problematic when used in isolation because it merely identifies underlying factor structure within observed variables without reference to outcome (i.e., construct coherence). Typically, confirmatory factor analysis (CFA) is generally required to test the appropriateness of the emergent model (Suhr, 2006). This is consistent with psychometric theorists, who contend that scale development should start with exploration (EFA) then progress to CFA. CFA is preferable when measurement models possess a well-developed underlying theory for hypothesized patterns of loadings (Hurley et al., 1997). In the case of BISS, Farias et al. (2013) advocate a single, general factor underpinning belief in science. Hence, a thorough examination of scale structure is required in order to establish the conceptual constraints of the scale and determine its usefulness as a general measure of belief in science.

The present study examined the psychometric properties of the BISS by performing two related studies. Study 1 evaluated the analysis performed by Farias et al. (2013) via utilizing Horn’s parallel analysis in addition to EFA. This was necessary to examine the replicability of Farias et al.’s (2013) results in an EFA context. Study 2 comprised a test of the resultant factor model from study 1 using CFA. Invariance testing followed an analysis of general factor structure, by assessing the degree to which different groups (males and females) performed on the measure. Invariance testing provides a further level of psychometric scrutiny by evaluating the extent to which scores reflect true differences across groups as opposed to artifacts of measurement bias (Brown, 2006; Byrne, 2010; Denovan et al., 2017a). Study 2 extended the preceding study by testing the emergent factor structure within an independent sample, and by assessing the convergent validity of BISS. Convergent validity is useful to assess whether a measure of a specific construct aligns with another measure it should theoretically relate to. The IPO-RT was an appropriate measure because it is a known correlate of belief in science, which indexes intuitive thinking. Specifically, the IPO-RT assesses proneness to reality testing deficits (Dagnall et al., 2014, 2015b, 2018). Explicitly, “the capacity to differentiate self from non-self, intrapsychic from external stimuli, and to maintain empathy with ordinary social criteria of reality” (Kernberg, 1996, p. 120). This delineation is consistent with Langdon and Coltheart’s (2000) information-processing style account of belief generation. Noting these conceptual features, researchers frequently use the IPO-RT as an index of experiential, intuitive thinking style (Drinkwater et al., 2012; Dagnall et al., 2015b; Denovan et al., 2017a).

## Materials and Methods

### Data Collection and Procedure

In order to evaluate the psychometric properties of the BISS two independent samples of respondents were required. To create these, amalgamation of data sets from previously published studies and ongoing research projects was undertaken. The researchers collected all data via online survey. In total, this comprised five merged data sets. Researchers have previously successfully utilized this method to generate large heterogeneous samples. Prominent examples are Revised Paranormal Belief Scale (Drinkwater et al., 2017), and Australian Sheep Goat Scale (Drinkwater et al., 2018).

Integration of BISS data sets was apposite since the research team have previously used the measure in comparable self-report studies. These have addressed a range of diverse research questions. The main advantage of data merging is the generation of sample sizes that permit the use of sophisticated statistical techniques. Explicitly, the combining data increases sample size, enhances statistical power and produces greater within sample variation (Van der Steen et al., 2008). This is particularly important when using procedures such as CFA, which require as many cases as possible (Brown, 2006). Hence, consolidation of BISS data was a convenient method that utilized existing, previously screened data to meet analytical constraints. Moreover, this approach generates a sample that would be difficult to recruit because of cost and time limitations.

Data collection for both studies occurred between September 2012 and September 2016 (see section “Ethics”). Recruitment was by emails to students (undergraduate and postgraduate) enrolled on healthcare programs (Nursing, Physiotherapy, Psychology, Speech, and Language Therapy, etc.), staff across faculties at the Manchester Metropolitan University, and local businesses/community groups. There were two exclusion criteria. Firstly, respondents had to be at least 18 years of age. Secondly, in order to prevent multiple responses instructions stated that respondents must not participate if they had undertaken similar or related research.

In all cases, respondents within the original research completed the BISS alongside several other measures. These assessed cognitive-perceptual personality factors, decision-making and anomalous beliefs (i.e., Irwin et al., 2015, 2016). In study 1, the BISS did not appear alongside the IPO-RT, whereas study 2 data derived from instances where the BISS and IPO-RT appeared within the same set of measures.

All studies employed the same, routine standardized procedures. Before undertaking the measures potential respondents received detailed information from the researchers. This outlined study aims, purpose, content, and ethical procedures. Assenting respondents provided informed consent via a survey option confirming willingness to participate. Subsequently, respondents received the study materials. Together with study measures there was a brief demographic section requesting age, preferred gender, and course of study if student, or occupation. Procedural instructions were consistent across studies. They directed respondents to progress through sections systematically, respond to items in an open and honest manner, work at their own pace, and reassured respondents that there were no right or wrong answers. To prevent potential order effects section order rotated across respondents.

### Ethics Statement

The research team gained ethical authorization for a program of studies exploring relationships between anomalous beliefs, decision-making and cognitive-perceptual personality factors as part of the grant bidding process. In total, there were three bi-annual calls (September 2012, 2014, and 2016). Review rated each application as routine and granted ethical approval. The Director of the Research Institute for Health and Social Change (Faculty of Health, Psychology and Social Care) and Ethics Committee within the Manchester Metropolitan University supervised this process. This process demanded that two experienced reviewers scrutinized the documentation. If research, as in this case, is classified as routine this constitutes full ethical approval. This was the required level of institutional approval at that point in time.

### Respondents

#### Study 1

The data set for study 1 contained 686 respondents. The mean (*M*) sample age was 26.70 years (*SD* = 11.07, range = 18–69 years). Disaggregation by gender revealed that 279 (40%) respondents were male and 407 (60%) female. Skewness and kurtosis values were within the recommended range of -2.0 to +2.0 (Byrne, 2010; Table 1). However, examination of multivariate normality suggested non-normality, as Mardia’s (1970) skewness (*b1p* = 9.80, *p* < 0.001) and kurtosis estimates (*b2p* = 29.737, *p* < 0.001) indicated significant deviation from a normal distribution.

**Table 1:** Summary statistics for all Study 1 and Study 2 variables.

	Study 1				Study 2								
BISS item	*M*	*SD*	Skew.	Kurt.	*M*	*SD*	Skew.	Kurt.	IPO-RT item	*M*	*SD*	Skew.	Kurt.
Q1	4.90	1.41	-1.38	1.09	5.21	1.27	-1.67	1.98	Q1	2.98	0.88	-0.19	0.04
Q2	4.05	1.46	-0.55	-0.48	4.73	1.44	-1.04	0.19	Q2	2.19	1.01	0.35	-0.79
Q3	3.97	1.53	-0.49	-0.82	4.27	1.67	-0.66	-0.85	Q3	2.47	0.97	0.22	-0.53
Q4	3.36	1.60	-0.03	-1.23	3.52	1.74	-0.17	-1.35	Q4	1.41	0.70	1.90	3.91
Q5	3.31	1.53	0.01	-1.09	3.65	1.69	-0.14	-1.25	Q5	2.43	0.97	0.15	-0.44
Q6	3.54	1.59	-0.15	-1.14	4.01	1.79	-0.47	-1.14	Q6	2.22	1.05	0.59	-0.32
Q7	3.19	1.60	0.17	-1.14	3.52	1.78	-0.08	-1.37	Q7	1.66	0.92	1.29	1.00
Q8	3.49	1.56	-0.12	-1.05	3.84	1.63	-0.37	-1.02	Q8	1.51	0.84	1.59	1.80
Q9	4.09	1.49	-0.65	-0.44	4.46	1.59	-0.89	-0.29	Q9	2.05	1.10	0.66	-0.64
Q10	4.57	1.32	-0.84	0.26	5.02	1.23	-1.18	0.73	Q10	2.14	0.96	0.48	-0.38
									Q11	1.71	0.85	1.07	0.68
									Q12	1.63	0.89	1.20	0.49
									Q13	1.93	0.91	0.85	0.46
									Q14	2.02	1.02	0.77	-0.18
									Q15	2.02	1.16	0.90	-0.18
									Q16	1.54	0.97	1.89	2.94
									Q17	2.26	1.07	0.29	-0.88
									Q18	1.71	0.88	1.12	0.63
									Q19	1.61	0.87	1.25	0.67
									Q20	2.13	1.01	0.68	-0.02


*BISS, Belief in Science Scale, IPO-RT, Inventory of Personality Organization-Reality Testing subscale.*

#### Study 2

The Study 2 sample comprised 534 (262, 49% male; 272, 51% female) respondents who had completed both the BISS and the IPO-RT. Mean (*M*) sample age was 37 (*SD* = 14.74, range = 18–71 years). All items, with the exception of IPO-RT items 4 and 16, demonstrated acceptable univariate skewness and kurtosis (i.e., between -2.0 and +2.0) (Table 1). Although, multivariate non-normality existed (skewness: *b1p* = 130.27, *p* < 0.001; kurtosis: *b2p* = 52.28, *p* < 0.001).

### Measures

#### Study 1

The only measure examined in Study 1 was the BISS. The BISS is a 10-item, self-report tool that assesses level of epistemic beliefs related to science. Specifically, items reference notions of scientific pre-eminence (i.e., the idea that science possesses unique and central value that provide a superior, exclusive guide to reality) (Farias et al., 2013; Valdesolo et al., 2016). Items take the form of statements (e.g., “We can only rationally believe in what is scientifically provable”), and respondents indicate level of agreement via a 6-point Likert scale (ranging from 1 “strongly disagree” to 6 “strongly agree”). Thus, raw scores range from 10 to 60, with higher scores indicating stronger belief in science. Previous work reports that the BISS is unidimensional and possesses high internal consistency (Farias et al., 2013; Irwin et al., 2015).

#### Study 2

In study 2, alongside the BISS, respondents completed the IPO-RT subscale of The Inventory of Personality Organization (IPO–RT; Lenzenweger et al., 2001). Within the IPO-RT, there are 20-items presented as statements (e.g., “When everything around me is unsettled and confused, I feel that way inside”). Respondents indicate the degree to which they endorse each statement using a five-point Likert scale (1 = never true to 5 = always true). Accordingly, total scores range from 20 to 100, with higher scores reflecting subjective evaluation of perceived likelihood of reality testing errors. Researchers often use IPO-RT scores as an index of intuitive thinking style (Denovan et al., 2017b). This derives from the supposition that the IPO-RT references suspension of reality testing, external critical evaluation (Irwin, 2004). Studies have established the psychometric properties of the IPO-RT. Particularly the measure possesses construct validity and demonstrates excellent internal consistency (α = 0.90; ω = 0.93) and test–retest reliability (Lenzenweger et al., 2001; Dagnall et al., 2018).

### Data Analysis

Psychometric examination of the BISS progressed through a series of increasingly sophisticated analytical techniques. These included Horn’s parallel analysis, exploratory factor analysis [EFA via maximum likelihood (MLR)], and CFA. The initial use of parallel analysis alongside scree plot assessment was necessary to judge the number of underlying factors. In addition, parallel analysis represents the most accurate approach to determine the quantity of factors to keep (Pallant, 2007). Accordingly, this included random resampling of the raw data (O’connor, 2000). EFA (SPSS 25) using the suggested number of factors then provided information on item loadings (Çokluk and Koçak, 2016).

Following parallel analysis and EFA, CFA conducted via Mplus 7.4 (Muthén and Muthén, 2015) assessed the appropriateness of data-model fit. Testing used the robust MLR method. This produces MLR parameter estimates and standard errors that are robust to instances of non-normality (Marsh et al., 2013).

The chi-square statistic (*χ^2^*), Comparative Fit Index (CFI), Tucker-Lewis Index (TLI) and absolute fit indices (Root-Mean-Square Error of Approximation, RMSEA; Standardized Root-Mean-Square Residual, SRMR) gaged model fit. The 90% confidence interval (CI) was included for RMSEA. CFI and TLI values >0.90 indicates good fit (Hopwood and Donnellan, 2010). According to Browne and Cudeck (1993), absolute values of 0.05, 0.06–0.08, and 0.08–1.0 reflect good, satisfactory, and marginal fit for RMSEA and SRMR.

Omega coefficient (estimated using JASP; Jeffreys’s Amazing Statistics Program) determined internal consistency before invariance testing. This is a more effective reliability estimate than popular approaches such as coefficient alpha, which typically over- or underestimates the true reliability of a measure (Deng and Chan, 2017). Multi-group CFA examined invariance of factor structure (configural), factor loadings (metric), and item intercepts (scalar) in relation to gender for the superior factor solution. Chen’s (2007) criteria of a CFI difference ≤ 0.01 and RMSEA ≤ 0.015 determined satisfactory fit for each invariance test.

In order to determine the replicability of the factor model from Study 1 in an independent sample, Study 2 analysis examined this model using CFA and measurement invariance. Also within Study 2, a test of convergent validity occurred. This involved comparing BISS with the criterion measure IPO-RT.

## Results

### Study 1

For parallel analysis, eigenvalues from the raw data with values higher than those from the random data represent the resultant factors. A parallel analysis (with 1000 resamples) revealed that one factor (eigenvalue = 5.64) possessed an eigenvalue higher than random data (eigenvalue = 1.19). Therefore, one factor existed. Scree plot assessment further confirmed this. EFA examined the BISS with the restricted number of factors (Çokluk and Koçak, 2016). Results revealed satisfactory sampling adequacy; Kaiser-Meyer-Olkin measure (KMO) = 0.92 and a reasonable item correlation matrix, Bartlett’s Test of Sphericity (*p* < 0.001). The single factor explained 56.43% of variance, and all factor loadings bar one (item 2) exceeded 0.4 (Norman and Streiner, 1994) with the majority of items (8 of 10) exceeding the strict factor loading requirements of 0.6 by Hair et al. (1998). Although item 2 loaded below 0.4, it exceeded the minimum cut-off of 0.32 suggested by Tabachnick and Fidell (2014). Lastly, examination of internal consistency revealed omega reliability was high for BISS, ω = 0.91.

### Study 2

A replication of the resultant one-factor model in study 1 with a separate dataset revealed (using CFA) good fit and marginal fit for RMSEA, *χ^2^* (35, *N* = 534) = 202.26, *p* < 0.001, CFI = 0.93, TLI = 0.91, RMSEA = 0.09 (90% CI of 0.08 to 0.10), SRMR = 0.04. Inspection of standardized parameter estimates (Table 2) reported a similar distribution of item loadings to study 1. Omega reliability was consistent with Study 1 (i.e., high for BISS, ω = 0.93). In addition, for IPO-RT omega reliability was good, ω = 0.88.

**Table 2:** Standardized parameter estimates for CFA in Study 2.

Item	Parameter estimate	*R^2^*
Q1 Science provides us with a better understanding of the universe than does religion.	0.59**	0.35
Q2 “In a demon-haunted world, science is a candle in the dark.” (Carl Sagan)	0.47**	0.22
Q3 We can only rationally believe in what is scientifically provable.	0.80**	0.65
Q4 Science tells us everything there is to know about what reality consists of.	0.79**	0.62
Q5 All the tasks human beings face are soluble by science.	0.82**	0.67
Q6 The scientific method is the only reliable path to knowledge.	0.90**	0.81
Q7 The only real kind of knowledge we can have is scientific knowledge.	0.88**	0.78
Q8 Science is the most valuable part of human culture.	0.76**	0.58
Q9 Science is the most efficient means of attaining truth.	0.81**	0.66
Q10 Scientists and science should be given more respect in modern society.	0.68**	0.46


*^∗∗^Indicate *p* < 0.001; all *R*^*2*^-values statistically significant at *p* < 0.001.*

Multi-group analysis comparing gender revealed good model fit at the configural level across indices (excluding RMSEA), *χ^2^* (70, *N* = 534) = 239.73, *p* < 0.001, CFI = 0.93, TLI = 0.91, RMSEA = 0.09 (90% CI of 0.08 to 0.10), SRMR = 0.04. For metric invariance, an acceptable CFI difference of 0.005 existed alongside a minimal RMSEA difference of 0.002. Scalar invariance testing indicated a satisfactory difference for CFI (0.009) and RMSEA (0.001).

A test of convergent validity examined Pearson correlations between total BISS with Reality Testing (IPO-RT). Total BISS possessed a significant negative correlation with IPO-RT, *r*(532) = -0.28, *p* < 0.001 (95% CI of -0.36 to -0.19). *Post hoc* analyses split BISS at the quartile level to assess further its relationship with IPO-RT. A one-way ANOVA (using bootstrapping with 1000 resamples) indicated a differential relationship existed between BISS quartiles and IPO-RT, *F*(3,530) = 17.62, *p* < 0.001. Given the identification of non-normality in the data, bootstrapping enables a more accurate estimation of *p*-values and standard errors (Byrne, 2010). Indeed, bootstrapping performs well even in datasets of extreme non-normality (Nevitt and Hancock, 2001), and is a suitable alternative to MLR estimation considering an ANOVA command is not present in Mplus. The bootstrapping procedure generated estimations of standard errors alongside bias-corrected and accelerated CIs (at the 95% confidence level). Further scrutiny via mean comparisons tested the possibility that the relationship between BIS and IPO-RT was not linear. Using Bonferroni correction revealed, that whilst no differences were present between the first and second quartile, scores above the median differed significantly from those below the median. This indicates that a moderate level of BISS is required before a decline in intuitive thinking becomes evident (Table 3).

**Table 3:** Reality testing scores as a function of belief in science quartiles.

	Comparisons (mean differences) between quartiles	
Contrast	Mean difference (*Sig.*)	95% BCa CI
Quartile 1 vs. Quartile 2	0.74 (0.537)	-1.58, 3.06
Quartile 1 vs. Quartile 3	4.99 (<0.001)**	2.49, 7.69
Quartile 1 vs. Quartile 4	7.75 (<0.001)**	5.39, 10.18
Quartile 2 vs. Quartile 3	4.25 (0.004)*	1.71, 6.85
Quartile 2 vs. Quartile 4	7.01 (<0.001)**	4.60, 9.35
Quartile 3 vs. Quartile 4	2.76 (0.026)*	0.48, 5.09


*^∗^Indicates *p* < 0.05, ^∗∗^indicates *p* < 0.001; 95% BCa CI: Bias-corrected and Accelerated confidence interval based on 1000 bootstrapped samples.*

## Discussion

The present paper found that, consistent with Farias et al. (2013), a one-factor solution best explained BISS scores. Further psychometric consideration revealed that the measure demonstrated good/excellent internal consistency across the two studies (study 1, ω = 0.91, study 2, ω = 0.93). Examination of scale items indicated that respondents esteemed both the principles of science (i.e., providing meaning) and the application of science to specific applications (i.e., problem solving).

Studies 1 and 2 validated the one-factor solution, signifying that this was congruent with the single factor model advocated by Farias et al. (2013). Support for the one-factor solution was compelling because study 2 using an independent sample replicated the model tested in study 1. In terms of convergent validity, the BISS negatively correlated with reality testing (*r* = -0.28). The size of this relationship was similar to the correlation observed by Irwin et al. (2015) (*r* = -0.32). Overall, findings suggest that belief in science is moderately associated with the tendency to engage in experiential, intuitive thought. Within the present study, the BISS correlated negatively with the IPO-RT.

Collectively study findings indicated that higher levels of belief in science were associated with a lower propensity to reality testing deficits. A caveat to this statement was the observation that a decline in RT scores was evident only within participants scoring above the median on BISS, *r* = -0.12, *n* = 269, *p* = 0.03 (95% CI of -0.02 to -0.24). Below the median, there was no relationship between BISS and IPO-RT, *r* = -0.01, *n* = 265, *p* = 0.449 (95% CI of -0.14 to 0.13). This implies that moderate levels of BISS were required to facilitate a reduction in subjective, experiential-based thinking.

This view is consistent with the conceptual nature of scientific thinking. Explicitly, that analytical thinking is a key tenet of the scientific approach. This includes critical evaluation in the form of rational enquiry and objective consideration of evidence. These features are inherently contrary to intuitive thinking, which draws upon experiential, subjective appraisal of information. In this context, the findings are congruent with Farias et al.’s (2013) notion that higher levels of belief in science reflect a preference for analytical thinking. This typically manifests as a predilection for objective, external fact based (vs. subjective experiential) evidence.

Although these conclusions are congruent with previous research, there are limitations to consider. A particular concern is the size of the correlation between BISS and RT, which was only in the medium range. Indeed, the variables shared only approximately 7% variance. This is indicative of the fact that a range of factors in addition to belief in science influence thinking style. These include, but are not restricted to, motivation or ability to expend cognitive effort (Shiloh et al., 2002), and ability, in the form of task-relevant background knowledge or expertise (Novak and Hoffman, 2008). Accordingly, future studies should examine the degree to which these factors interact with belief in science. It seems likely that high (vs. low cognitive) load and level of proficiency will influence the degree to which individuals appraise information, make decisions and draw on faith in science. With hindsight, the observation of a small correlation concurs with the view that the IPO-RT assesses a peculiar definition of thinking style. Specifically, one that indexes reality distortions and psychotic like phenomena (Lenzenweger et al., 2001).

A further concern is that both the BISS and IPO-RT are only “proxy” indirect measures of preferential thinking style. Accordingly, the scales do not directly assess thought. Instead, they index qualities reflective of the respective thinking style (Denovan et al., 2017b). In this context, it is important to note that BISS assesses “belief in the veracity of the scientific principles and methods,” and IPO-RT taps the inclination to draw upon internal (rather than external) cognitions. Moreover, the present study failed to consider demographic factors such as level of education and occupational statues, which may indirectly influence critical thinking and belief in science. Thus, subsequent research could consider also the degree to which these factors affect belief in science.

Regarding BISS, there is an important distinction between confidence in the concept of science and the application of science based rationality. Many scientific informed discussions, such as those around climate change and the extinction of the dinosaurs, require systematic evaluation of information collected via methodical means. However, this process is often truncated, or terminated prematurely. This is often the case when individuals hold strong views about a topic and select (either consciously or unconsciously) evidence that supports their perspective. This assimilation bias leads to the dismissal of disconfirming evidence (Lord et al., 1979; Whitmarsh, 2011). Hence, it is possible to have a high belief in science, but base decision making on experiential (intuitive) rather than rational (analytical) appraisal of evidence.

In the case of the IPO-RT, reality testing is an abstract, spontaneous cognitive-perceptual process. Subsequently, individuals may lack either conscious awareness, or veridical insight into the nature of reality testing (Denovan et al., 2017b). This is especially true because metacognition encompasses two principle mechanisms, knowledge of and control of cognition (Larkin, 2009; Schneider and Artelt, 2010). Measuring cognitive processes is difficult for these reasons. This is true of metacognitive measures generally. Consequently, the relationship between subjective performance and actual performance is often weak (Rabbitt and Abson, 1990; Reid and MacLullich, 2006; Buelow et al., 2014). Hence, future studies should examine the extent to which belief in science predicts performance on objective critical thinking skills tests. This will reveal the degree to which belief in the scientific approach corresponds to an analytical thinking style.

It would also be worthwhile examining interactions between other factors related to cognitive style, such as dogmatism, and belief in science. Dogmatism is particularly pertinent because it denotes close-mindedness (Rokeach, 1960; Shearman and Levine, 2006). Specifically, the propensity to select and process information in a manner that reinforces prior opinions/expectations (Ottati et al., 2018). Accordingly, inflexible adherence to belief is likely to affect appraisal of evidence independent of thinking style. Open-minded cognition in contrast is unbiased and involves selection and processing of information in a manner unaffected by prior opinions/expectations (Church and Samuelson, 2016; Ottati et al., 2018). In the case of belief in science, this could produce overreliance on the concept of science and a dismissal of the limitations of the scientific approach. This is certainly the case when science acts as a form of faith that assists individuals to cope with stressful and anxiety-provoking situations (Farias et al., 2013). This represents an affective rather than a rational approach, which is the antithesis of analytical, objective thought. Hence, scientific extremism is a form of radical secular faith characterized by a subjective worldview.

This paper indicates that the BISS is satisfactory at a psychometric level. However, further research is necessary because belief in science is a relatively new construct. Explicitly, consideration of this alongside other belief related measures would further understanding of the belief in science construct. This is important because secular beliefs, such as Humanism and belief in progress have demonstrated the same compensatory mechanisms as belief in science (Rutjens et al., 2010). Examining relationships between these factors will provide a better understanding of their commonalities and differences. For instance, belief in science provides a framework for comprehending the world. Within this science, people may regard science as intellectually and socially progressive. However, science in the strictest sense is neutral and amoral.

Indeed, as Sarewitz (2015) notes, the social, moral, and ethical implications of deploying advances, such as new technology are contentious rather than the science findings. Thus, scientific advancements may not produce beneficial outcomes. In this context, it may prove worthwhile to investigate whether increased understanding of the scientific method reduces its positive effects relative to Humanism and belief in progress. If no differences are evident, then this suggests that any belief system that provides explanations of the world will afford comfort and assurance (see Preston and Epley, 2005). Thus, it may be that positive beliefs by their nature have beneficial psychological effects. These arise largely from subjective rather than evidential means.

## Ethics Statement

The research team gained ethical authorization for a program of studies exploring relationships between anomalous beliefs, decision-making, and cognitive-perceptual personality factors as part of the grant bidding process. In total, there were three bi-annual calls (September 2012, 2014, and 2016). Review rated each application as routine and granted ethical approval. The Director of the Research Institute for Health and Social Change (Faculty of Health, Psychology and Social Care) and Ethics Committee within the Manchester Metropolitan University supervised this process. This process demanded that two experienced reviewers scrutinized the documentation. If research, as in this case, was classified as routine this constitutes full ethical approval. This was the required level of institutional approval at that point in time.

## Author Contributions

ND contributed to theoretical focus and analysis, and design, background, and data collection. AD contributed to theoretical focus, and led on analysis and model testing. KD contributed to and supported all sections. AP commented on drafts – provided theoretical background and draft feedback.

## Conflict of Interest Statement

The authors declare that the research was conducted in the absence of any commercial or financial relationships that could be construed as a potential conflict of interest.
